# Characteristics of Neonicotinoids in Colostrum from Shanghai, China (2007–2019): Concentration Levels, Temporal Trends, and Potential Health Risk

**DOI:** 10.3390/toxics13050366

**Published:** 2025-05-01

**Authors:** Kexin Li, Minghui Fu, Bingli Lei, Xiuhua Shen, Xinyu Zhang, Jun Xu, Xiaolan Zhang

**Affiliations:** 1 School of Environmental and Chemical Engineering, Shanghai University, Shanghai 200444, China; echo_lkx@163.com (K.L.); fmh2817@163.com (M.F.); leibingli@126.com (B.L.); 2 School of Public Health, Hongqiao International Institute of Medicine, Shanghai Jiao Tong University School of Medicine, Shanghai 200025, China; srachel@126.com; 3 Department of Clinical Nutrition, Xinhua Hospital Affiliated to Shanghai Jiao Tong University School of Medicine, Shanghai 200025, China; 4 Department of Clinical Nutrition, College of Health Science and Technology, Shanghai Jiao Tong University School of Medicine, Shanghai 200025, China; xyzhang999@shsmu.edu.cn; 5 Shanghai Second People’s Hospital, Shanghai 200011, China; 13901658489@163.com

**Keywords:** neonicotinoids, colostrum, temporal variation, health risk assessment

## Abstract

Neonicotinoids (NEOs) are widely used neuroactive insecticides with several adverse effects on human health. This study examined 186 colostrum samples collected at three time points between 2007 and 2019 from Shanghai, China to investigate the distribution and temporal variations of NEOs. The median total concentration (ΣNEOs) was 136 ng/L, with the imidacloprid equivalent concentration (IMIeq) of 249 ng/L. N-desmethyl-acetamiprid (DM-ACE) had the highest median level at 49.6 ng/L, accounting for 43.9% of ΣNEOs, followed by imidacloprid (IMI) (20.1 ng/L and 22.1%). Thiamethoxam (THM), clothianidin, and acetamiprid were also identified as important parent compounds (p-NEOs). Temporal variations suggested a decrease in ΣNEOs, IMIeq, and DM-ACE concentrations from 2013 to 2019; however, the total concentrations of p-NEOs remained comparable. Distinct trends were also observed in the concentrations of dinotefuran and IMI. Maternal body mass index and weight changes, which reflect the dietary habits of mothers, appeared to influence IMI and THM levels. No statistically significant relationships were found between colostrum concentrations and birth parameters using full-term birth data in 2019. The estimated hazard quotients (≤0.003), which were far below the risk threshold of 1, generally indicated negligible health risks for breastfeeding neonates. Nevertheless, the substantial contribution from several p-NEOs warrants further investigation.

## 1. Introduction

Neonicotinoids (NEOs), a category of synthesized neuroactive insecticides, have been extensively utilized since the 1990s, and currently represent one of the most widely used categories of insecticides [1,2]. The synthesis of imidacloprid (IMI) in the early 1990s marked the beginning of this family; then, the family expanded to include other compounds such as thiamethoxam (THM), clothianidin (CLO), and dinotefuran (DIN) [3]. With their widespread application, NEOs are frequently found in multiple environmental matrices, including surface water and numerous food types [4,5]. These compounds are also found in various human biological samples, such as blood, breast milk, urine, and hair [6,7,8].

The popularity of NEOs is due to their broad spectrum of activity and high efficiency [2]. Nevertheless, a large number of toxicological studies have suggested potential adverse impacts on non-target species [9,10]. Thus, the European Union (EU) has imposed restrictions on the use of IMI, CLO, and THM in specific crops and outdoor environments [11]. Meanwhile, the United States Environmental Protection Agency (USEPA) has reached provisional conclusions aimed to reduce the ecological risks [12]. Epidemiological studies have also associated NEO exposure with neurological disorders and reproductive system symptoms in mammals [10,13]. Notably, early exposure in infants has been linked to elevated risks of congenital anomalies, malformations, and psychiatric disorders [14]. Prenatal exposure to IMI or acetamiprid (ACE) was observed to associate with smaller head circumferences in newborns [15]. Additionally, a study in the USA also indicated that frequent exposure of mothers during pregnancy was significantly correlated with an increased risk of congenital heart defects in their children [16]. Given that neonatal exposure to NEOs can occur through placental transfer followed by breastfeeding, there is particular concern regarding potential health risks posed to newborns [14,17,18].

As a vital biological matrix, breast milk provides a direct evaluation of cumulative exposures in lactating mothers and their breastfed infants [7,17]. Previous research monitored NEOs in breast milk, with the total concentrations (ΣNEOs) ranging from a few to several thousand ng/L [7,14,17,18,19]. The parent NEOs (p-NEOs), including IMI, THM, CLO, and ACE, are usually identified as the main contaminants in breast milk [7,18,19]. And n-desmethyl-acetamiprid (DM-ACE) has been suggested as an important pollutant in breast milk across 23 provinces in China [17]. Overall, there is a limited number of studies about NEO metabolites in breast milk, and regional differences in NEO contamination have been consistently observed [17,20]. For example, concentration levels in Asia were relatively higher compared to those in USA [17,21,22]. And a Chinese study involving 3570 participants found that ΣNEOs in milk samples from southern China were higher than those from northern areas [17]. Several prior studies have indicated time-related changes in urinary NEO concentrations among the general population [23,24], and variation in milk pollution levels over time has been reported in only one recent Chinese study [7]. They proposed that THM gradually replaced DM-ACE as the primary contaminant in breast milk from Guangzhou, China from 2014 to 2022 [7]. Colostrum is a type of breast milk produced in the first few days after childbirth. Few studies have been conducted to examine colostrum NEO levels and their potential temporal variations [17,18].

We hypothesized that the presence of NEOs in colostrum may exhibit temporal fluctuations due to usage restrictions, and these variations could differ across various regions in China. To examine the potential characteristics of temporal variation in eastern China, a total of 186 colostrum samples were collected in three batches (2007, 2013, and 2019) from Shanghai, China. The concentrations of NEOs and their temporal variations were subsequently analyzed. Furthermore, we examined demographic factors that may influence maternal exposures, as well as the possible effects on birth parameters of neonates.

## 2. Materials and Methods

### 2.1. Standards

Six standards, including IMI, ACE, THM, CLO, thiacloprid (THCP), and DIN, were acquired from Anpel Laboratory Technologies (Shanghai) Inc. (Shanghai, China). DM-ACE, IMI-d_4_, and ACE-d_3_ were acquired from Dr. Ehrenstorfer (Augsburg, Germany). Additionally, 1-methyl-3-(tetrahydro-3-furylmethyl) urea (DIN-U), 5-hydroxy-imidacloprid (5-OH-IMI), olefin-imidacloprid (IMI-Of), DIN-d_3_, and THCP-d_4_ were acquired from Alta Scientific Co., Ltd. (Tianjin, China), THM-d_3_ from A ChemTek_b_ Inc. (Worcester, MA, USA), and CLO-d_3_ from Toronto Research Chemicals (Toronto, ON, Canada) was also purchased.

### 2.2. Sampling and Information Collection

Human colostrum samples (n = 186) were obtained from postpartum volunteers at three distinct periods between 2007 to 2019. Those samples were collected within the initial postpartum period (3–5 days after delivery) from two urban hospitals in Shanghai, China. Participants met predefined inclusion criteria: continuous residency in Shanghai for more than 3 years and no history of occupational pesticide exposure. Recruitment was conducted randomly by gynecologists and midwives at the time of delivery. All samples were immediately stored at −20 °C prior to laboratory processing. While bias due to sample selection cannot be ruled out, all samples and data were blinded to treatment. Ethics committees were provided by both participating hospitals and written informed consent was secured from all volunteers.

Demographic data, including maternal age and educational background, were obtained via questionnaires during sample collection. Clinical records provided additional parameters such as maternal anthropometric measurements (weight and height), neonatal sex, and birth metrics (weight and length). The study population comprised 189 infants: 89 in 2019 (including two pairs of fraternal twins and a pair of boy–girl twins), 52 in 2013, and 48 in 2007 (Table 1). In the survey, the birth parameters were not collected in 2007, and head circumference information was missing for neonates in 2010. And for neonates in 2019, some specific information such as birth weight (n = 2) and birth length (n = 28) was lost, involving a total of 32 samples. Maternal BMI (kg/m^2^) was determined by dividing body weight by the square of height. The ponderal index (PI, g/cm^3^) was calculated as the ratio of birth weight to the cube of birth length [15].

### 2.3. Sample Treatment Protocols

A modified method from a previous report was employed to extract NEOs [17]. Each colostrum sample (5 mL) was spiked with internal standards (2.5 ng each, including IMI-d_4_, ACE-d_3_, THM-d_3_, CLO-d_3_, THCP-d_4_, and DIN-d_3_), followed by protein precipitation and extraction using acetonitrile. The supernatant was concentrated and then transferred to QuEChERS d-SPE purification tubes (50 mg PSA, 50 mg C18, 150 mg MgSO_4_, 2 mL) (Shimadzu, Kyoto, Japan). The eluate was concentrated and reconstituted using specific volumes of acetonitrile. The resulting solution was then analyzed using HPLC-MS/MS.

### 2.4. Instrumental Analysis

The target compounds were analyzed using an LC-Agilent Technologies 1290 Infinity HPLC-MS/MS (MS-AB SCIEX QTRAP 6500; Milford, MA, USA). A Poroshell C18 column (150 × 2.1 mm, 2.7 μm, Agilent) was used. The mobile phase, consisting of water with 0.1% formic acid (A) and acetonitrile (B), was programmed accordingly with a flow rate of 0.4 mL/min. Positive ion mode electrospray ionization (ESI^+^) was used for mass spectrometry. Multiple reaction monitoring mode (MRM) was used for quantification. Specific parameters of MRM transitions alongside instrumental conditions are documented (Table S1).

### 2.5. Estimated Daily Intake and Health Risk Assessment of Exposure to NEOs

To assess the total exposure to NEOs, which comprise both p-NEOs and their metabolites, the imidacloprid equivalent concentration (IMIeq) was calculated using the following equation:

(1) $${IMI}_{eq}=\sum ({RPF}_{i}\times C_{i})\;=2.9\times C_{DIN}+5.8\times C_{CLO}+14.2\times C_{THCP}+1.0\times C_{IMI}+9.5\times C_{THM}\;+0.8\times C_{ACE}+0.8\times C_{DM-ACE}+1.0\times C_{5-OH-IMI}\;+1.0\times C_{Of-IMI}+2.9\times C_{DIN-U}$$

where *C* (ng/L) is the concentration of target species. *RPFi* (dimensionless) is the relative potency factor for each corresponding NEO, standardized to IMI. The RPF values were obtained from a previous reference [25].

To compare the potential health risks associated with individual NEO compounds, hazard quotients (HQ, dimensionless) were used and calculated as follows:

(2) $$HQ=\frac{EDI}{RfD}$$

where EDI (ng/kg bw/day), the abbreviation for estimated daily intake, is calculated by multiplying the colostrum concentration by the time of milk consumption and then dividing by the neonate’s body weight. It is assumed that a neonate weighing 6 kg ingested nearly 0.75 L/day of milk during the first week after birth [7]. RfD is the chronic reference dose (Table S2) [26,27]. An HQ value of 1 or above indicates an obviously health risk [28].

### 2.6. Quality Assurance and Quality Control

To avoid potential contamination, multiple measures were implemented in the laboratory. For each batch, two procedural blanks were conducted along with 12–15 samples, and no blank values were found. Internal standards (ISs) were used throughout all experiments. To test the extraction efficiency of the method, matrix spiking experiments were conducted. When the spiked concentration was 50 ng/mL, the average IS-corrected recoveries ranged from 60.3 ± 7.0% (DIN-U) to 130.1 ± 7.5% (IMI) (n = 4–6). The method detection limits (LODs) were defined as six times the corresponding standard deviation, ranging from 0.3 to 3.9 ng/L (Table S3). And the method quantification limits (LOQs) were set at two times the level of LODs.

### 2.7. Statistical Analysis

Statistical analysis was conducted using IBM SPSS Statistics software (Version 23.0). Differences between samples were analyzed using the Mann–Whitney *U* test, the Kruskal–Wallis *H* test, one-way ANOVA, independent samples *t*-test, or chi-square test as appropriate. *p* < 0.05 was the threshold for a statistically significant difference. Measured concentrations below LOD were treated as zero in statistical analysis. If the concentration exceeded LOD but was below LOQ, it was considered as a LOQ/√2.

## 3. Results and Discussion

### 3.1. Characteristics of Study Populations

The anthropometric data from the survey are presented (Table 1). The average age of mothers varied across the three sampling periods. Notably, the maternal ages in both 2019 and 2013 were obviously different from those in 2007 (*p* < 0.05). The values of maternal body mass index before pregnancy (pBMI) were 17.6–29.4 kg/m^2^, and the values of maternal body mass index before delivery (aBMI) were from 21.1 to 38.0 kg/m^2^. The male-to-female ratio of newborns was 80:59. And in 2019, there was a loss of gender information, along with two pairs of fraternal twins and one pair of boy–girl twins. The averaged birth weight and birth height were 3160 ± 596 g and 49.2 ± 1.8 cm, respectively. And the averaged PI was (2.7 ± 0.3) × 10^–2^ g/cm^3^ (Table 1). There were also significant differences in birth weights and birth lengths between the 2019 and 2013 samples (*p* < 0.05).

### 3.2. Occurrence and Distribution Patterns

The compounds that were frequently detected (>75% detection frequencies) included DM-ACE, THM, IMI, CLO, and ACE (Table S4). IMI-Of, 5-OH-IMI, DIN, and DIN-U were found in 30.6–56.5% of all colostrum samples, while THCP was sporadically detected. Four parent compounds (p-NEOs), IMI, THM, CLO, and ACE, were all detected in 148 out of 186 samples, indicating the widespread presence of NEOs in colostrum from Shanghai, China.

**Figure 1:**
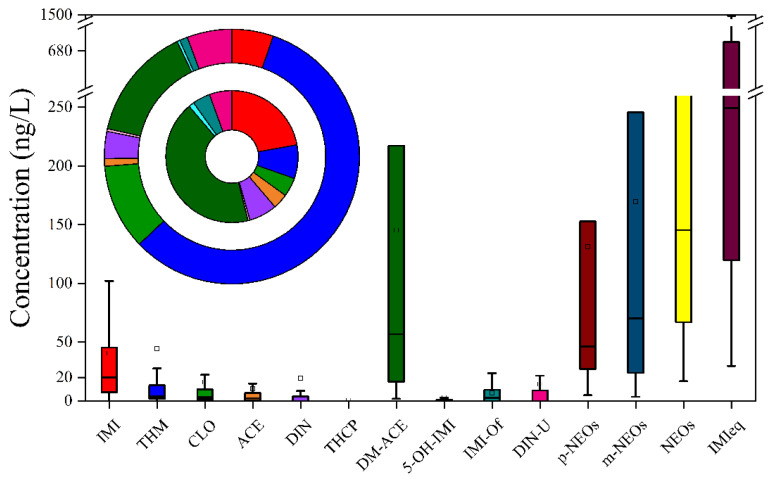
Concentrations (box plot) and distribution profiles (circle graph) of neonicotinoids in colostrum. IMIeq: imidacloprid equivalent concentration. In the box plot graph, box plots depict the interquartile range, with the central line and square symbol representing the median and mean values. Vertical whiskers indicate the 5th and 95th percentile thresholds for data distribution. In the circle graph, the outer ring indicates the distribution percentages of IMIeq, while the inner ring represents the proportion of the total concentrations.

Among the three sampling periods, the total concentration ranges of NEOs (including their metabolites, ∑NEOs) were between 16.8 and 1.63 × 10^3^ ng/L (median: 136 ng/L), while IMIeq ranged from 29.7 to 6.31 × 10^3^ ng/L (median: 249 ng/L) (Figure 1). The median level of IMIeq was nearly two that times of ∑NEOs. DM-ACE (a primary metabolite of ACE) and IMI were the two top pollutants, with the median concentrations of 49.6 and 20.1 ng/L, respectively. THM, CLO, and ACE were also important parent compounds in colostrum, owing to the high detection frequencies and comparable concentrations (medians: 1.9–3.8 ng/L). The concentration of IMI-Of, a kind of metabolite, exhibited a relatively high value (median: 7.2 ng/L), but lower detection frequency (56.5%) than those of THM, CLO, and ACE. The total concentration of metabolites (∑m-NEOs) (median: 76.1 ng/L, mean: 219 ng/L) was significantly higher, compared to the total concentration of p-NEOs (∑p-NEOs, median: 46.0 ng/L, mean: 160 ng/L) (Table S4). Additionally, substantial concentration variabilities were usually observed, such as ∑NEOs and IMIeq, indicating obviously different individual contaminations in colostrum samples.

Several studies have reported Σp-NEOs in China [7,14,17]. The total concentrations of 6–8 parent compounds ranged from 38.2 ng/L to 387 ng/L (22–137 individual samples, and 97 pool samples from 3570 mothers) [14,17,19]. The ∑p-NEOs in the present study had a comparable range but were much higher than concentrations reported from Switzerland, USA, and India [20,29,30]. IMI was usually reported as the most abundant p-NEO [14,19]. However, the descending concentration order of other p-NEOs in our study (ACE > THM > CLO) was different from a few previous studies (THM > ACE > CLO, or CLO > THM > ACE) [7,14,17]. In addition, m-NEOs were detected in several Chinese studies, where DM-ACE consistently dominated [14,17], a phenomenon also found in the present study. The occurrence of NEOs in colostrum indicated that neonates would intake p-NEOs and their metabolites through breastfeeding.

For the composition profile, DM-ACE was identified as the predominant m-NEO, while IMI emerged as the dominant p-NEO in colostrum, accounting for 43.9% and 22.1% of ∑NEOs, respectively (Figure 1). The concentration percentage of IMI was nearly two to five times greater than that of THM, CLO, ACE, and DIN (3.7–8.2%), whereas the percentage of DM-ACE was approximately an order of magnitude higher compared to other m-NEOs (from 1.4% to 5.5%). Additionally, THM and CLO contributed relatively more to IMIeq than to ∑NEOs (12.3–29.0% vs. 4.6–8.2%) (Figure 1).

Following systemic uptake, p-NEOs undergo primary metabolic transformation via phase I enzymatic pathways, specifically cytochrome P450. In healthy adults, ACE exhibits rapid hepatic conversion to its principal metabolite DM-ACE, achieving complete biotransformation within 48 h [31]. In human milk collected from China, DM-ACE was frequently detected at high percentage, and often accompanied by IMI or THI [7,14,17]. For example, Chen et al. reported distribution profiles of NEOs in human milk from China during the period of 2017−2019, suggesting that DM-ACE accounted for an average of 61.2% of ∑NEOs, followed by IMI at 15.6%. And the averaged percentage of DM-ACE was approximately eight times higher than that of its parent form ACE [17]. Consistent with these findings, our study also revealed a significantly higher concentration percentage of DM-ACE (43.9%) compared to ACE (3.7%) in colostrum samples. The p-NEOs is a class of neuro-active insecticides, which was suggested to pass through human placenta and influence fetal and infant health [32,33]. DM-ACE is also suggested to have detrimental effects on serum testosterone levels and semen quality [34,35]. The high concentration percentages of DM-ACE, THM, and CLO in colostrum underscore the need for monitoring of both parent NEOs and their metabolites in human milk.

### 3.3. Time Variations

**Figure 2:**
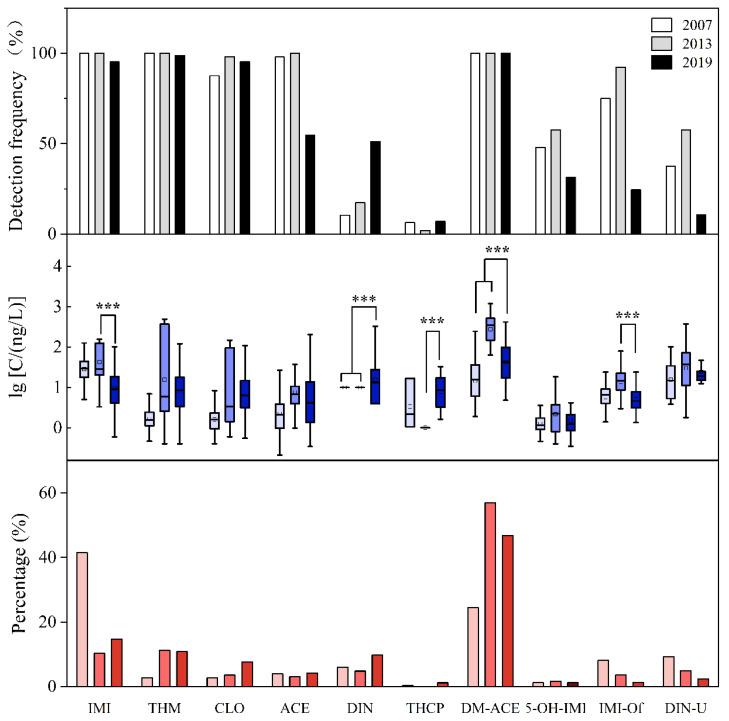
Time variations in detection frequencies, concentrations (Log-transformed), and distribution percentages. Box plots display the interquartile range, with a horizontal line denoting the median and a central marker indicating the mean value. Vertical whiskers extend to the 5th and 95th percentiles, defining the data distribution range. ***: Mann–Whitney *U* test, *p* < 0.001.

Obvious temporal variations were observed during the sampling periods from 2007 to 2019. The highest ΣNEOs and IMIeq were found in a colostrum sample collected in 2013, and these levels declined significantly from 2013 to 2019 (Mann–Whitney *U* test, *p* < 0.001). The median ∑NEOs in 2013 (589 ng/L) was nearly five times higher than that in 2019 (100 ng/L) (Table S4). Colostrum concentrations of DM-ACE also decreased from 346 ng/L in 2013 to 42.6 ng/L in 2019 (Figure 2). Although the Σp-NEOs in 2019 was comparable to those in 2013 (44.3 ng/L vs. 49.3 ng/L), parent compounds exhibited notable yet distinct variations from 2007−2013 to 2019. DIN concentrations significantly increased (Kruskal–Wallis *H* test, *p* < 0.001), along with an increase in detection frequencies (from 10.4%−17.3% to 51.2%) and a consistent level of concentration percentages (4.8−6.0% in 2007−2013 vs. 9.8% in 2019). Conversely, IMI concentrations decreased from the elevated levels observed in 2013 to those in 2019 (Mann–Whitney *U* test, *p* < 0.001), yet they maintained similar concentration percentages over this period (10.3% in 2013 compared to 14.6% in 2019) (Figure 2).

Studies examining the temporal variation in NEO contamination in human matrices are limited. Huang et al. conducted an analysis of human milk samples from Guangzhou, China, over the period from 2014 to 2022 (5 samples per year from 2014 to 2018, and 25−32 samples in 2019−2022) [7]. The data indicated a statistically significant decrease in concentrations of total NEOs, DM-ACE, and ACE between 2014 and 2022. However, increasing trends for CLO and THM levels were observed during the same time period (*p* < 0.01) [7]. Ueyama et al. examined changes in urinary NEO concentrations among Japanese women from 1994 to 2011 [24]. They observed a notable rise in the detection frequencies of urinary NEOs, which suggested high exposure to NEOs during that period in Japan [24]. The colostrum concentration variations were also observed in our study. In addition, in a Chinese food study, the detection frequency of DIN increased during the period of 2015–2018, compared to 2009–2012, and IMI concentrations in various food categories were usually lower in 2015–2018 [36]. Given that dietary intake is a main or important exposure way [36], the increase in both concentration and detection frequency in colostrum DIN in our study was consistent with food contamination trends.

Global regulatory actions have escalated in response to ecological concerns. The EU banned outdoor uses of IMI, CLO, and THM in 2018 [11], while the USEPA proposed mandatory mitigation measures for ACE, CLO, DIN, IMI, and THM in 2022 [12]. These actions consequently resulted in changes in usage patterns, as well as changes in environmental presence and human exposure [3,24]. NEOs are registered in more than 120 countries, comprising nearly 24% of the global pesticide market, with sales peaking around 2010 [37]. The observation of the highest levels of colostrum NEOs in 2013, along with a subsequent significant decline in present study, matched this trend. IMI was the first registered NEO in 1991. Following that, CLO and THM were successively introduced in China after the year 2000 [2]. Additionally, DIN has been approved for use in China since 2013 [8] and has had a significant increase in its use over the past decade [3]. A similar shift in DIN and THM levels was also observed in colostrum samples from Shanghai, China. The observed change in NEO contaminations raised significant concerns regarding the potential risks associated with multiple exposures to NEOs through breastfeeding. It is important to note that the sample sizes in the present study were limited, and the maternal ages and educational backgrounds across the three sampling periods varied significantly. These factors may influence the extent of colostrum contamination and potentially affect temporal changes. Further investigations into milk contamination are necessary to ascertain whether this trend exists and how it evolves over time.

### 3.4. Associations of Demographic Characteristics

To assess the potential impacts of demographic data, we classified mothers into subgroups based on pBMI, aBMI, or gestational weight gain (GWG). The subgroups included a thin (pBMI < 18.5, n = 2 and 7 in 2019 and 2013, respectively), normal (18.5 ≤ pBMI ≤ 24.0, n = 54 and 33 in 2019 and 2013, respectively), and obese group (pBMI > 24.0, n = 17 and 12 in 2019 and 2013, respectively), or low (aBMI < 25th percentile, n = 19 and 13 in 2019 and 2013, respectively), median (25th ≤ aBMI ≤ 75th, n = 40 and 25 in 2019 and 2013, respectively), and high group (aBMI > 75th; n = 21 and 14 in 2019 and 2013, respectively). For GWG, participants were categorized according to the Chinese standard [38], which included inadequate/excessive group (n = 40 and 23 in 2019 and 2013, respectively), and recommend group (n = 30 and 29 in 2019 and 2013, respectively) (Table S4).

**Figure 3:**
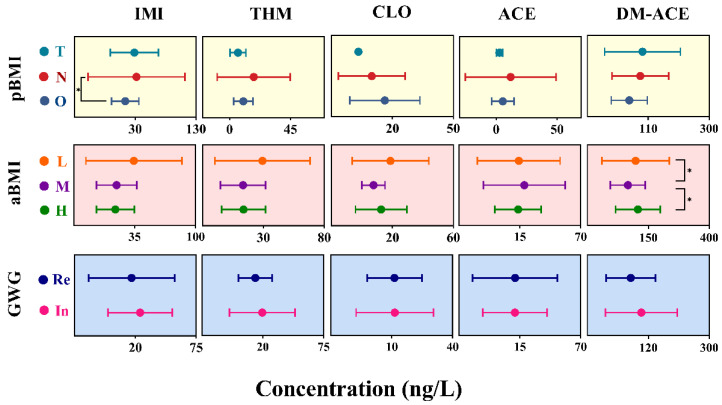
Associations between colostrum concentrations (in 2019) and maternal weight parameters. *: Kruskal–Wallis *H* test, *p* < 0.05. pBMI: maternal body mass index before pregnancy; aBMI: maternal body mass index before delivery; GWG: gestational weight gain. Group T: thin pBMI subgroup of mothers (pBMI < 18.5, n = 2); Group N: normal pBMI subgroup of mothers (18.5 ≤ pBMI ≤ 24.0, n = 54); Group O: obese pBMI subgroup of mothers (pBMI > 24.0, n = 17). Group L: low aBMI subgroup of mothers (aBMI < 25th percentile, n = 19); Group M: median aBMI subgroup of mothers (25th ≤ aBMI ≤ 75th, n = 40); Group H: high aBMI subgroup of mothers (aBMI > 75th percentile, n = 21); Group Re: recommended GWG subgroup of mothers, including mothers who gained weight during pregnancy in accordance with recommended standards, n = 40; Group In: inadequate or excessive GWG subgroup of mothers, including mothers who gained inadequate or excess weight during pregnancy, n = 30.

Obvious group differences in the colostrum concentrations of individual NEOs were frequently observed in 2019 (Figure 3). Although these concentration differences were often not statistically significant, mothers with normal pBMI exhibited higher colostrum levels of IMI or THM, compared to the obese group. And mothers who gained the recommended weight during pregnancy had low colostrum levels of IMI or THM (Figure 3). Additionally, mothers in the low aBMI group generally displayed relative high colostrum concentrations, while comparable concentrations were often found between the medium and high aBMI groups. Similar phenomena were also observed in 2013 (Table S5). Overall, GWG and BMI may affect colostrum concentrations, which are often associated with maternal dietary habits before and during pregnancy.

**Figure 4:**
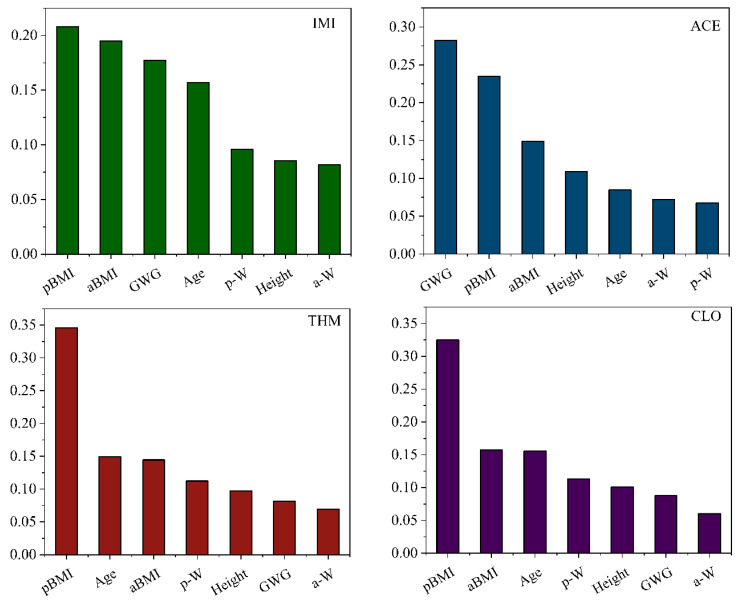
A Random Forest simulation of the important influencing factors. aBMI: maternal body mass index before delivery; pBMI: maternal body mass index before pregnancy; GWG: gestational weight gain. p-W: maternal weight before pregnancy; a-W: maternal weight before delivery. The combined data in 2019 and 2013 was used.

Random Forest simulations were conducted to rank influential factors using combined data from 2013 and 2019. The testing results were favorable for p-NEOs with high detection frequencies (>75%) with the training R-value exceeding 0.84. Generally, aBMI and pBMI were among the top three important variables influencing the concentrations of IMI, THM, CLO, and ACE. For example, pBMI was the primary influence factor on IMI, followed by aBMI and GWG. while pBMI, and aBMI were the first and third important variables for THM (Figure 4). The influence of BMI on the concentrations of most p-NEOs also suggested a possible relation with maternal dietary habits.

The possible influence of maternal weights and other demographic data was also analyzed in several studies involving pregnant women [6,39]. A prior investigation conducted in Hangzhou, China (n = 137) found a significant difference in milk IMI concentrations when maternal BMI was considered [19]. The mean IMI levels in the underweight (BMI < 18.5), normal (18.5 < BMI < 24.9), and overweight mothers (BMI ≥ 25) were reported as 68.6, 32.6, and 47.7 ng/L, respectively [19]. Furthermore, a study from Wenzhou, China (n = 432) observed that NEOs and ACE+DM-ACE showed more frequent detection in urine samples from pregnant women who were overweight before pregnancy (pBMI ≥ 24) [40]. Additionally, a significant association between inadequate GWG during pregnancy and lower urine concentrations of desnitro-imidacloprid was observed in a study of China (n = 3289) [6]. Food consumption is considered as an important factor influencing NEO exposures [39,41]. Higher intake frequencies of both plant-based and animal-derived foods were correlated with increased detection frequencies of urinary NEOs in pregnant women [42]. The present study indicated that BMI and GWG may affect colostrum concentrations, which may be largely linked to maternal dietary habits before and during pregnancy. Pregnant women have increasing nutritional requirements to support fetal growth, highlighting the need for further research into how maternal nutrition influences NEO levels in colostrum.

### 3.5. Influence of Colostrum Exposure on Birth Parameter

To assess potential prenatal exposure effects on birth parameters, colostrum, which reflects the late-pregnancy maternal body burden, was analyzed for its correlation with birth weights and infant PI. The colostrum concentrations in 2019 showed no significant correlations with these parameters in full-term infants (fetal age above 37 weeks) (multivariable linear regression, *p* = 0.089–0.988) (Table 2). Stratifying the data by infant sex did not suggest any modifications in the statistical data. IMI and DM-ACE exhibited negative effects on birth weights (standardized β coefficients: −0.076 and −0.165), while ACE and THM showed positive effects (standardized β coefficient: 0.025 and 0.180), though these effects were not statistically significant (Table 2). A similar phenomenon was observed in the influence of PI values. The possible influence of individual NEOs seemed to be different.

Few studies provided epidemiological data on the impact of prenatal NEO exposure. For example, a cohort study involving 296 mother–infant pairs from Laizhou Wan, China between 2010 and 2013 suggested that prenatal IMI exposure (based on maternal urinary concentrations) was positively associated with infant PI; however, negative correlations were observed between prenatal exposure to IMI (or ACE) and neonatal head circumference among all infants [15]. In a nest case–control study conducted in China (n = 1483), maternal blood concentrations of ACE and DIN were associated with an elevated risk of fetal growth restriction, as determined by a non-linear model (*p* < 0.05) [32]. However, the potential influence on birth weight or PI values in our study was not significantly evident. Our study was constrained by a limited sample size (n = 47–65), and the statistical data need to be carefully interpretated. In our opinion, the current understanding of prenatal exposure’s influence on fetal growth remains inadequate. The use of diverse human matrices for estimating exposure levels, variations in sampling periods across different pregnancy stages, and differing sampling sizes would lead to contradict effects that may reach statistical significance [43,44]. Further epidemiological research is needed for better understanding.

### 3.6. Health Risk Assessment

The EDI was calculated based on the aforementioned assumption of body weight and milk consumption. Under medium and high-exposure scenarios (95%), the total EDI values in 2019 ranged from 15.04 to 82.77 ng/kg bw/day. When parent compounds and the related metabolites were considered together, the EDI values for ACE and its metabolite DM-ACE (ACE_T_, median: 8.25 ng/kg bw/day; high: 47.85 ng/kg bw/day) were always the highest, followed by DIN+DIN-U (DIN_T_, 16.97 ng/kg bw/day) and IMI+IMI-Of+5-OH-IMI (IMI_T_, 16.44 ng/kg bw/day) in conditions of high exposure.

**Figure 5:**
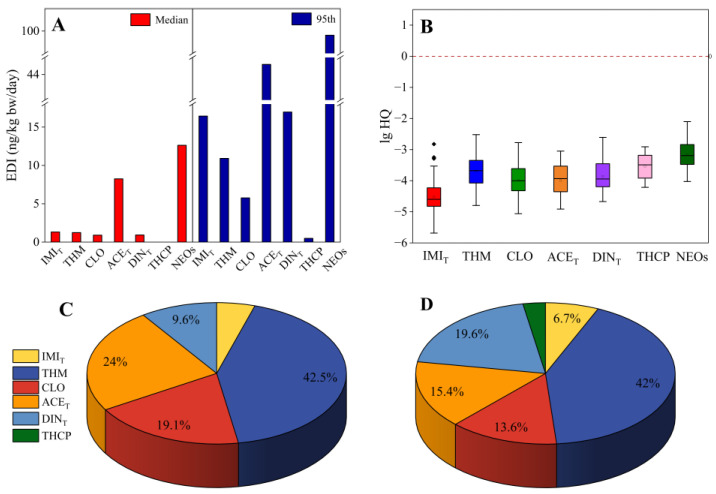
Estimated daily intake of neonates in the first week of life through breastfeeding (**A**), hazard quotient (**B**), percentage contributions to the overall health risk under medium exposure scenarios (**C**), and under high-exposure scenarios (**D**). IMI_T_: including IMI, 5-OH-IMI, and IMI-of; ACE_T_: including ACE and DM-ACE; DIN_T_: including DIN and DIN-U. Boxes in (**B**) represent interquartile ranges; the square and central line within each box denote the median and mean levels, respectively; upper and lower whiskers indicate the 95th and 5th percentiles, respectively.

The highest HQ value was 0.003 for THM. The averaged HQ values were consistently three to five orders of magnitude lower than the health risk threshold (HQ = 1) (Figure 5). Therefore, the overall health risks for neonates from breastfeeding were negligible. Nevertheless, THM contributed to 42–42.5% of the total health risk, while the percentage contribution of DIN_T_ increased from 9.6% in a medium exposure scenario to 19.6% in a high-exposure scenario (Figure 5). Infants are particularly vulnerable to environmental toxins during their early developmental stages. Early postnatal exposure would have significant long-term health consequences for infants, such as an increased risk of childhood obesity and reduced Full-Scale IQ scores by age seven [14,44,45]. The substantial contribution of THM and DIN_T_ to potential health risk highlights the urgent need for serious attention in this area.

## 4. Conclusions

Four parent compounds and one metabolite were prevalent in colostrum, with the median ΣNEOs amounting to half of the IMIeq. Between 2007−2013 and 2019, there was a general change in NEO concentrations. Specifically, the concentrations of total NEOs, IMIeq, IMI, and DM-ACE exhibited decreasing trends from 2013 to 2019. Conversely, both the DIN level and detection frequency showed an increasing pattern during this period. This trend was likely attributable to changes in usage patterns. Dietary habits during and before pregnancy may influence maternal weight changes, thereby affecting NEO levels in colostrum. Concentrations of primary NEO pollutants were not statistically significantly related to the birth weight or ponderal indexes in 2019, though the statistical analysis had a limited sample size.

The HQ values were three to five orders of magnitude lower than the established health risk threshold (HQ = 1). This generally suggested no obvious health risk associated with breastfeeding. However, the notable contributions of THM and DIN_T_ are of concern, particularly in light of the recent increase in DIN concentrations. Furthermore, the number of NEO metabolites was few in the present study. Enhanced epidemiological studies and further investigation into the potential impact of multiple NEO exposures are warranted.

## Figures and Tables

**Table 1:** Maternal and neonatal anthropometric data and maternal educational background [mean ± SD or N (%)].

Characteristic	2019 (n = 86)	2013 (n = 52)	2007 (n = 48)	All (n = 186)
*Mothers*				
Age (years)	30.0 ± 3.3 ^#^	29.2 ± 3.5 ^#^	27.7 ± 3.6	29.2 ± 3.5
≤30	59 (68.6)	34(65.4)	32 (66.7)	125
>30	27 (31.4)	18 (34.6)	16 (33.3)	61
pBMI (kg/m^2^)	22.1 ± 2.7	22.0 ± 2.8	/	22.1 ± 2.6
<25	62 (72.1)	32 (61.5)	/	94
≥25	11 (12.8)	8 (15.4)	/	19
Information lost	13 (15.1)	12 (23.1)	/	73
aBMI (kg/m^2^)	27.3 ± 2.8	28.0 ± 3.7	/	27.6 ± 3.2
<25	19 (22.1)	10 (19.2)	/	29
≥25	61 (71.0)	37 (71.2)	/	98
Information lost	6 (6.9)	5 (9.6)	/	59
Education				
Under college	11 (12.8) ^&^	19 (36.5)	/	30
College and above	66 (76.7) ^&^	20 (38.5)	/	86
Information lost	9 (10.5)	13(25.0)	/	70
*Infants*				
Boys	53 (59.6) ^c^	27 (51.9)	/	80 ^c^
Girls	34 (38.2) ^c^	25 (48.1)	/	59 ^c^
Information lost	2 (2.3)	0	/	50
Fetal age				
less than 37 weeks	21 (23.6)	/	/	21
over 37 weeks	67 (75.3)	/	/	67
Information lost	1 (1.1)	/	/	101
Birth weight (g)	3080 ± 645 ^&^	3390 ± 409	/	3160 ± 596
Infants	87 (97.8)	52 (100)	/	139
Information lost	2 (2.2)	0	/	50
Birth length (cm)	48.5 ± 2.2 ^&^	50.1 ± 0.5	/	49.2 ± 1.8
Infants	61 (68.5)	52 (100)	/	113
Information lost	28 (31.5)	0	/	76
Ponderal index	0.027 ± 0.003	0.027 ± 0.003	/	0.027 ± 0.003
Infant	57 (64.0)	52	/	109
Information lost	32 (36.0)	/	/	80

pBMI: maternal body mass index before pregnancy; aBMI: maternal body mass index before deliveries; ^c^: including two pairs of fraternal twins and a pair of boy–girl twins. ^#^: statistically different from the data in 2007; ^&^: statistically different from the data in 2013. One-way ANOVA, independent samples *t*-test, or chi-square test was used to analyze differences, with statistical significance set at *p* < 0.05.

**Table 2:** The potential influence of colostrum concentrations (ng/L) on birth weight (kg) and ponderal index (g/cm^3^) of neonates using multiple linear regression analysis.

	Boys		Girls		All	
	β (95%CI)	*p*-Value	β (95%CI)	*p*-Value	β (95%CI)	*p*-Value
Birth weight (n = 65, including 35 boys and 30 girls)						
IMI	−2.28 (−11.97, 7.42)	0.612	2.44 (−4.43, 9.31)	0.454	−0.59 (−3.92, 2.74)	0.721
THM	−5.67 (−22.01, 10.69)	0.458	2.67 (−24.05, 29.38)	0.831	2.67 (−7.59, 12.93)	0.600
CLO	9.04 (−20.03, 38.11)	0.504	3.95 (−24.01, 31.91)	0.763	0.09 (−12.17, 12.35)	0.988
ACE	−12.7 (−38.0, 12.5)	0.288	−1.67 (−9.14, 5.80)	0.635	0.19 (−3.00, 3.39)	0.903
DM-ACE	−0.09 (−2.26, 2.09)	0.932	−1.79 (−4.44, 0.87)	0.168	−0.55 (−1.82, 0.71)	0.380
Ponderal index (n = 47, including 25 boys and 22 girls)						
IMI	−0.02 (−0.08, 0.03)	0.360	0.01 (−0.03, 0.04)	0.770	−0.02 (−0.04, 0.003)	0.089
THM	−0.05 (−0.15, 0.04)	0.243	0.02 (0.13, 0.17)	0.793	−0.02 (−0.08, 0.04)	0.485
CLO	0.08 (−0.09, 0.25)	0.315	0.03 (−0.13, 0.18)	0.723	0.05 (−0.03, 0.12)	0.198
ACE	0.003 (−0.03, 0.03)	0.859	−0.02 (−0.06, 0.02)	0.378	0.004 (−0.02, 0.02)	0.656
DM-ACE	−0.01 (−0.02, 0.01)	0.371	−0.01 (−0.03, 0.004)	0.133	−0.01 (−0.01, 0.002)	0.165

The multiple linear regression models included adjustments for maternal age and pre-pregnancy body mass index (pBMI). The analyses of the infant-sex subgroup were further adjusted for all the aforementioned confounders, excluding gender. Data from full-term births in 2019 were used for analysis. IMI: imidacloprid; THM: thiamethoxam; CLO: clothianidin; ACE: acetamiprid; DM-ACE: n-desmethyl-acetamiprid.

## Data Availability

Data will be made available on request.

## Supplementary Materials

The following supporting information can be downloaded at: https://www.mdpi.com/article/10.3390/toxics13050366/s1. Table S1: Optimized MS/MS parameters for neonicotinoids and internal standards; Table S2: Reference doses (RfD) of neonicotinoids; Table S3: The method detection limits, quantification limits, and matrix spike recoveries of neonicotinoids; Table S4: Concentrations of neonicotinoids in colostrum samples (ng/L); Table S5: Average neonicotinoid concentrations (standard deviations) among different subgroups of mothers (ng/L); References [26,27,46,47,48,49] are cited in Supplementary Materials.
