# Supplementary material for: Characteristics of Neonicotinoids in Colostrum from Shanghai, China (2007–2019): Concentration Levels, Temporal Trends, and Potential Health Risk
Source: Toxics. 2025 May 1;13(5):366. doi: 10.3390/toxics13050366 (PMC12115666; doi:10.3390/toxics13050366)
Supplement: Supplementary file 1 [file toxics-13-00366-s001.zip › toxics-3552278-supplementary.pdf]

Characteristics of neonicotinoids in colostrum from Shanghai, China (2007–2019): Concentration levels, temporal trends, and potential health risk

Kexin Li[1], Minghui Fu[1], Bingli Lei[1], Xiuhua Shen[2 3], Xinyu Zhang[4], Jun Xu[5], Xiaolan Zhang[1*]

[1] School of Environmental and Chemical Engineering, Shanghai University, Shanghai, 200444, China

[2] School of Public Health, Hongqiao International Institute of Medicine, Shanghai Jiao Tong University School of Medicine, Shanghai, 200025, China

[3] Department of Clinical Nutrition, Xinhua Hospital Affiliated to Shanghai Jiao Tong University School of Medicine, Shanghai, 200025, China

[4] Department of Clinical Nutrition, College of Health Science and Technology, Shanghai Jiao Tong University School of Medicine, Shanghai, 200025, China

[5] Shanghai Second People's Hospital, Shanghai, 200011, China

* Correspondence to: Prof. Xiaolan Zhang, E-mail: zhangxiaolan@shu.edu.cn

1. Instrumental analysis

The target compounds were analyzed by an LC-Agilent Technologies 1290 Infinity HPLC-MS/MS (MS-AB SCIEX QTRAP 6500; Milford, MA, USA). To separate the chemicals, a Poroshell C18 column (150 × 2.1 mm, 2.7 μm, Agilent) was used. Water with 0.1% formic acid (A) and acetonitrile (B) were used as the mobile phase with a flow rate of 0.4 mL/min. The mobile phase was programmed and started at 2% B, increased to 60% B over 3.6 min, and to 95% B over 0.2 min, then held for 1.5 min at 95% B. The injection volume was 2 μL. Multiple reaction monitoring mode (MRM) and electrospray ionization in the positive ion mode were used for quantification. Details of MRM transitions and instrumental conditions are listed in **Table S1**.

2. Tables

Table S1 Optimized MS/MS parameters for neonicotinoids and internal standards.

Compound	Parent ion	Product ions	CE (eV)	DP (eV)	EP (eV)	CXP (eV)
IMI	256	209*, 175	19 (26)	60 (55)	9 (7)	8 (6)
THM	292	211*, 181	15 (28)	58 (52)	6	8 (6)
CLO	250	169*, 132	20 (19)	54	6 (5)	10
ACE	223	126*, 56	28 (22)	63 (67)	9 (12)	9
DIN	203	129*, 114	16 (15)	2 (3)	4 (3)	8 (7)
THCP	253	126*, 90	29 (48)	95 (97)	6 (10)	9 (8)
DM-ACE	209	126*, 90	23 (42)	52 (57)	10	9 (14)
5-OH-IMI	272	225*, 191	20 (25)	63 (66)	3	7 (8)
IMI-Of	254	171*, 205	22 (20)	55 (52)	3	12 (11)
DIN-U	159	85*, 67	20 (25)	47 (48)	10	6 (4)
IMI-d_4	260	179*, 213	28 (20)	81 (80)	9	6 (8)
THM-d_3	295	214*, 184	16 (28)	54 (50)	6 (5)	15
CLO-d_3	253	172*, 132	14 (19)	55	3	6 (11)
ACE-d_3	226	126*, 90	27 (46)	86 (82)	10 (9)	9 (6)
DIN-d_3	206	132*, 116	16 (15)	56 (50)	2	4
THCP-d_4	257	126*, 90	30 (53)	95 (90)	7 (4)	9 (6)

*: quantitative ion; CE: collision energy; DP: declustering potential; EP: entrance potential; CXP: collision cell exit potential.

Table S2 Reference doses (RfD) of neonicotinoids.

Compounds	RfD (ng/kg bw/day)	Compounds	RfD (ng/kg bw/day)
IMI	5.70×10^4	THCP	4.00×10^3
THM	6.00×10^3	DM-ACE	7.10×10^4
CLO	9.80×10^3	5-OH-IMI	5.70×10^4
ACE	7.10×10^4	IMI-Of	5.70×10^4
DIN	2.00×10^4	DIN-U	2.00×10^4

*: Obtained from References [1-6].

Table S3 The method detection limits, quantification limits, and matrix spike recoveries of neonicotinoids.

Compound	LODs (ng/L)	LOQs (ng/L)	Matrix spike recovery (mean ± SD) (%)		
			5 ng	10 ng	50 ng
IMI	0.6	1.1	141.7±8.5	143.6±11.2	131.2±3.3
THM	0.4	0.8	126.9±1.6	127.6±6.4	108.4±2.3
CLO	0.6	1.1	121.6±7.2	119.3±7.8	107.8±9.1
ACE	0.3	0.6	102.2±1.8	105.0±3.5	85.6±0.1
DIN	3.9	7.9	136.0±2.8	136.4±14.9	126.8±1.1
THCP	0.3	0.6	117.7±2.2	113.1±3.1	108.2±3.3
DM-ACE	0.5	0.9	105.8±6.0	101.0±4.1	89.6±1.4
5-OH-IMI	0.4	0.7	52.5±5.4	73.7±7.8	54.8±7.0
IMI-Of	1.4	2.7	85.7±4.4	93.6±13.5	83.0±6.5
DIN-U	0.9	1.8	51.9±8.7	66.5±4.3	71.3±14.3

LODs: method detection limits, calculated based on six times the corresponding standard deviation; LOQs: the method quantification limits, LOQ=2LOD; SD: standard deviation (n = 4−6).

Table S4 Concentrations of neonicotinoids in colostrum samples (ng/L)

	IMI	THM	CLO	ACE	DIN	THCP	Σp-NEOs	DM-ACE	5-OH-IMI	IMI-Of	DIN-U	Σm-NEOs	∑NEOs	IMIeq
All (n=186)														
DF (%)	97.8	99.5	94.1	78.5	31.2	5.4		100	43.0	56.5	30.6			
Median (mean)	20.1(53.9)	2.4 (62.5)	1.9 (19.2)	3.8 (12.2)	<LOD (12.5)	<LOD (5.09)	46.0 (160)	49.6 (185)	<LOD (3.72)	7.2 (10.4)	<LOD (20.6)	76.1 (219)	136 (380)	249 (795)
Range	<LOD-209	<LOD-493	<LOD-149	<LOD-148	<LOD-220	<LOD-16.8	4.9-797	1.9-1.19×10^3	<LOD-195	<LOD-79.7	<LOD-380	3.6-1.19×10^3	16.8-1.63×10^3	29.7-6.31×10^3
Percentage (%)	22.1	8.2	4.6	3.7	6.8	0.5	46.1	43.9	1.4	4.3	5.5	53.9	/	/
2019 (n=86)														
DF (%)	95.3	98.8	95.3	54.7	51.2	7.0		100	31.4	24.4	10.5			
Median (mean)	8.8 (25.0)	8.2 (16.9)	5.7 (11.1)	<LOQ (8.92)	<LOQ (17.7)	<LOD (0.86)	44.3 (80.5)	42.6 (82.4)	<LOD (2.27)	<LOD (1.94)	<LOD (3.43)	55.6 (90.0)	100 (171)	244 (400)
Range	<LOD-547	<LOD-121	<LOD-109	<LOD-207	<LOD-330	<LOD-32.7	5.5-660	4.8-420	<LOD-117	<LOD-54.7	<LOD-122	4.8-421	20.9-897	35.6-3.00×10^3
Percentage (%)	14.6	10.9	7.7	4.2	9.8	1.1	48.3	46.8	1.3	1.3	2.4	51.7	/	/
2013 (n=52)														
DF (%)	100	100	98.1	100	17.3	1.9		100	57.7	92.3	57.7			
Median (mean)	28.6 (67.6)	6.1 (129)	3.2 (37.5)	6.8 (16. 5)	<LOD (23.4)	<LOD (0.02)	49.3 (251)	346 (352)	<LOQ (6.07)	13.9 (16.4)	6.5 (37.0)	412 (411)	589 (686)	809 (2.01×10^3)
Range	3.3-159	0.4-493	<LOD-149	0.7-138	<LOD-297	<LOD-1.01	9.3-797	63.1-1.19×10^3	<LOD-195	<LOD-79.7	<LOD-380	71.7-1.19×10^3	81.0-1.63×10^3	74.7-6.31×10^3
Percentage (%)	10.3	11.2	3.5	3.1	4.8	0.0	32.8	57.0	1.7	3.5	4.9	67.2	/	/
2007 (n=48)														
DF (%)	100	100	87.5	97.9	10.4	6.3		100	47.9	75.0	37.5			
Median (mean)	29.3 (38.9)	1.6 (1.86)	1.4 (2.10)	2.1 (6.41)	<LOD (20.3)	<LOD (4.17)	37.7 (67.5)	15.8 (35.3)	<LOD (0.81)	4.8 (5.80)	<LOD (9.71)	27.5 (50.8)	84.1 (118)	121 (192)
Range	2.4-209	<LOQ-6.9	<LOD-17.1	<LOD-148	<LOD-220	<LOD-16.8	4.9-435	1.9-538	<LOD-3.6	<LOD-24.1	<LOD-103	3.6-538	16.8-974	29.7-1.28×10^3
Percentage (%)	41.5	2.7	2.8	3.9	6.0	0.3	57.1	24.4	1.2	8.1	9.2	42.9	/	/

DF: detection frequency. <LOD: below the method detection limit; <LOQ: below the method quantification limits.

Table S5 Average neonicotinoid concentrations (standard deviations) among different subgroups of mothers (ng/L)

	IMI	THM	CLO	ACE	DM-ACE
pBMI					
2019 <18.5 (n=2)	28.7 (39.1)	6.24 (5.86)	3.25 (0.20)	2.67 (2.61)	51.0 (56.3)
18.5-24.0 (n=54)	32.1 (79.8)	17.9 (27.1)	9.82 (16.4)	11.9 (37.3)	84.9 (88.3)
>24.0 (n=17)	13.6 (22.4)*	10.2 (7.20)	16.2 (17.3)	5.46 (9.19)	92.0 (118)
2013 <18.5 (n=7)	78.2 (56.7)	227 (210)*	65.0 (59.1) *	9.68 (12.3)	346 (156)
18.5-24.0 (n=33)	68.3 (56.6)	133 (200)	39.2 (56.2)	15.9 (28.6)	268 (209)
>24.0 (n=12)	59.7 (56.6)	60.2 (136)*	17.1 (36.9) *	22.0 (23.2)	399 (303)
aBMI					
2019 <25th (n=19)	34.1 (51.2)	29.2 (39.1)	18.7 (25.2)	14.0 (47.2)	107 (91.7)*
25th-75th (n=40)	15.6 (21.6)	13.3 (18.6)	7.56 (7.59)	5.30 (12.8)	66.2 (71.8)
>75th (n=21)	14.4 (20.3)	13.7 (17.9)	12.6 (16.9)	14.0 (37.3)	97.9 (138)*
2013 <25th (n=13)	80.4 (61.3)	229 (220)	64.9 (61.6)	19.0 (36.8)	390 (160)
25th-75th (n=25)	63.9 (54.1)	105 (181)	30.5 (50.5)	13.4 (21.0)	305 (210)
>75th (n=14)	62.4 (55.9)	82.5 (160)	24.8 (45.8)	19.7 (22.1)	418 (320)
GWG					
2019 Inadequate (n=40)	24.4 (28.9)	19.6 (29.6)	11.5 (19.1)	10.5 (28.9)	99.4 (106)
Recommend (n=30)	16.7 (38.8)	13.2 (15.1)	11.3 (13.5)	10.6 (38.3)	68.2 (72.7)
2013 Inadequate (n=23)	75.2 (61.3)	141 (211)	40.5 (58.5)	21.5 (32.0)	417 (255)
Recommend (n=29)	61.7 (51.3)	120 (178)	35.2 (50.6)	12.5 (19.0)	305 (189)

pBMI: body mass index before pregnancy; aBMI: body mass index before delivery; GWG: gestational weight gain. Inadequate: subgroup including mothers who gained inadequate or excess weight during pregnancy. Recommend: subgroup including mothers who gained weight during pregnancy in accordance with recommended standards. *: significant difference compared to normal subgroup of mothers ($p < 0.05$), the normal subgroup of mothers is 18.5≤ pBMI ≤ 24.0, or 25th≤ aBMI ≤ 75th, or GWG recommend.

References

1. US Environmental Protection Agency, 2012. Clothianidin; pesticide tolerance. https://www.federalregister.gov/documents/2012/08/29/2012-21215/clothianidin-pesticide-tolerances. (accessed on 5 April 2025).
2. US Environmental Protection Agency, 2013a. Dinotefuran; pesticide tolerance. https://www.federalregister.gov/documents/2013/04/10/2013-08400/dinotefuran-pesticide-tolerances. (accessed on 5 April 2025).

3. US Environmental Protection Agency, 2013b. Imidacloprid; pesticide tolerances for emergency exemptions. https://www.federalregister.gov/documents/2013/06/05/201313203/imidacloprid-pesticide-tolerances. (Accessed 1 May 2020).
4. US Environmental Protection Agency, 2013c. Thiacloprid; pesticide tolerances. https://www.federalregister.gov/documents/2013/02/06/2013-02692/thiacloprid-pesticidetolerances. (accessed on 5 April 2025).
5. US Environmental Protection Agency, 2017. Thiamethoxam; pesticide tolerance. https://www.federalregister.gov/documents/2017/02/15/2017-03075/thiamethoxam-pesticide-tolerance. (accessed on 5 April 2025).
6. US Environmental Protection Agency, 2020. Acetamiprid; pesticide tolerances. https://www.federalregister.gov/documents/2020/02/14/2020-02038/acetamiprid-pesticide-tolerances. (accessed on 5 April 2025).
